# Pregnancy Outcomes in Women with Thyroid Diseases

**DOI:** 10.31662/jmaj.2021-0191

**Published:** 2022-02-28

**Authors:** Tomomi Kotani, Kenji Imai, Takafumi Ushida, Yoshinori Moriyama, Tomoko Nakano-Kobayashi, Satoko Osuka, Hiroyuki Tsuda, Seiji Sumigama, Eiko Yamamoto, Fumie Kinoshita, Akihiro Hirakawa, Akira Iwase, Fumitaka Kikkawa, Hiroaki Kajiyama

**Affiliations:** 1 Department of Obstetrics and Gynecology, Nagoya University Graduate School of Medicine, Nagoya, Japan; 2 Centre for Maternal-Neonatal Care, Nagoya University Hospital, Nagoya, Japan; 3 Department of Obstetrics and Gynecology, Fujita Health University School of Medicine, Toyoake, Japan; 4 Department of Obstetrics and Gynecology, Japanese Red Cross Nagoya Daiichi Hospital, Nagoya, Japan; 5 Office of International Affairs/International Medical Education, Nagoya University Graduate School of Medicine, Nagoya, Japan; 6 Department of Healthcare Administration Nagoya University Graduate School of Medicine, Nagoya, Japan; 7 Data Science Division, Data Coordinating Center, Department of Advanced Medicine, Nagoya University Hospital, Nagoya, Japan; 8 Department of Biostatistics and Bioinformatics, Graduate School of Medicine, The University of Tokyo, Tokyo, Japan; 9 Department of Obstetrics and Gynecology, Gunma University Graduate School of Medicine, Maebashi, Japan

**Keywords:** hyperthyroidism, hypothyroidism, placental abruption, preeclampsia

## Abstract

**Introduction:**

Overt hyperthyroidism and hypothyroidism are associated with pregnancy complications; however, most women with these conditions are diagnosed before conception and are under treatment during pregnancy, especially in high-income countries. The purpose of this study was to investigate pregnancy complications among these women.

**Methods:**

A retrospective cohort study was conducted, and data on pregnant women who gave birth to a singleton at Nagoya University Hospital in Japan in 2005-2014 was collected. The pregnancy outcomes were divided and compared among three groups: the control group (n = 3531), the hyperthyroidism group (n = 48), and the hypothyroidism group (n = 61). Additionally, risk factors for placental abruption were evaluated by multivariable logistic regression analysis. Moreover, in hyperthyroidism, thyroid function at the placentation period was compared between placental-related diseases and nonplacental-related disease groups, and the latter group included placental abruption and preeclampsia.

**Results:**

The incidence of placental abruption was higher in hyperthyroidism than in control and hypothyroidism groups. Hyperthyroidism was independently associated with an increased risk of placental abruption (adjusted odds ratio, aOR = 8.21, 95% confidence interval, CI: 1.76-38.34), as well as preeclampsia (aOR = 4.10, 95% CI: 1.13-14.76) and preterm labor (aOR = 3.38, 95% CI: 1.19-9.64). Additionally, thyroid-stimulating hormone (TSH) at the placentation period was significantly lower in the placental-related disease group than in the nonplacental-related disease group (*p* < 0.05).

**Conclusions:**

Pregnancy outcomes in women with hyperthyroidism and hypothyroidism would be comparable with those without thyroid disease. Hyperthyroidism was an independent risk factor for placental abruption as well as preterm labor and preeclampsia. However, its frequency was extremely low, and further research is required to validate our findings.

## Introduction

Thyroid diseases, including hyperthyroidism and hypothyroidism, are common disorders in women of childbearing age. When left untreated, they can have adverse effects on maternal and neonatal outcomes. A large cohort study demonstrated that primary hypothyroidism was associated with increased pregnancy complications, including preeclampsia, gestational diabetes, preterm birth, induction of labor, and cesarean section ^(1)^. It also revealed that hyperthyroidism was associated with preeclampsia, preterm birth, and induction of labor. A meta-analysis reported that both overt hypothyroidism and hyperthyroidism were associated with an increase in preterm birth ^(2)^. Most women in developed countries with hyperthyroidism or hypothyroidism are diagnosed and treated before conception, and only a few studies have reported the pregnancy outcomes in such cases ^(3)^. Thus, before and during pregnancy, the diagnosis and treatment of thyroid disease are recommended to minimize adverse outcomes ^(4)^. Previous reports on population-based and case-control studies demonstrated that hyperthyroidism is associated with a high risk of preeclampsia ^(5), (6), (7), (8)^ and placental abruption ^(9)^. However, the impact of interventions for hyperthyroidism before and during pregnancy on pregnancy outcomes remains uncertain ^(10)^, although one study reported a higher occurrence of adverse pregnancy complications in an untreated hyperthyroidism group than a treated group ^(11)^. Therefore, even though most women are already on medication, the effect of treated hyperthyroidism or hypothyroidism on pregnancy outcomes remains unknown.

In the present study, we investigated the pregnancy outcomes of women with thyroid disease who had been diagnosed and treated before their pregnancy: birth weight, cesarean section, Apgar scores, and obstetric complications including preterm labor, preeclampsia, light for dates (LFD) infants, premature rupture of membranes (PROM), placenta previa, placenta abruption, and preterm birth. We also focused on placental abruption, the leading cause of maternal and neonatal mortality and morbidity.

## Materials and Methods

### Statement of ethics

The study was approved by the ethics committee of Nagoya University Graduate School of Medicine (2015-0415, 2017-0414). This study was a retrospective study by the ethical guidelines of the Japanese Ministry of Health, Labor, and Welfare. Thus, the requirement for informed consent was waived by the ethics committee of Nagoya University Graduate School of Medicine. However, patients had been informed on the hospital website and provided an option to opt out from their medical records being used in research.

### Study participants

**Figure 1.**
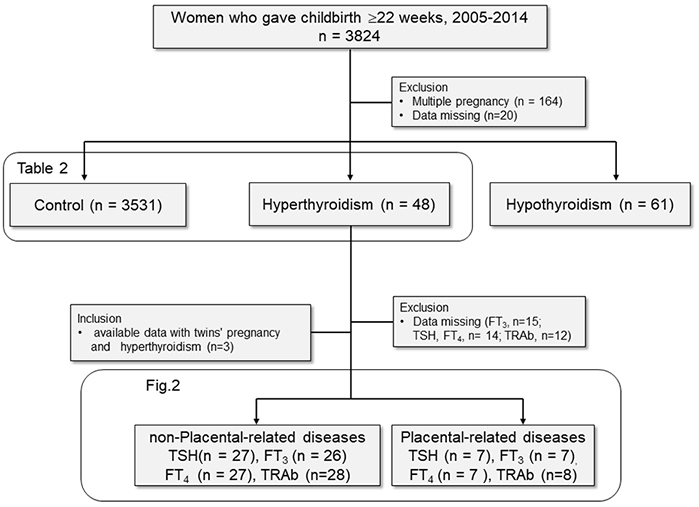
Flow chart of the study subjects. Pregnancy outcomes were compared among the control, hyperthyroidism, and hypothyroidism groups. Risk factors of placental abruption were determined via univariate and multivariate analyses in the control and hyperthyroidism groups (Table 2). Additionally, the association between thyroid function and placentation was examined in the hyperthyroidism group (Figure 2): the nonplacental-related disease group vs. the placental-related disease group.

The study samples were retrospectively recruited from medical records at a single tertiary care institute, Nagoya University Hospital in Japan. The inclusion criteria for this study were women who gave birth at Nagoya University Hospital located in Japan from 2005 to 2014 (Figure 1, n = 3824). Additionally, patients who treated thyroid diseases at other hospitals were included when their treatment information from the hospital was provided. The exclusion criteria for this study were as follows: multiple pregnancies (n = 164) and incomplete medical records (n = 20). Subjects were divided into three groups, namely, the control group: those without thyroid disease (n = 3531); the hyperthyroidism group: those who were diagnosed and treated for hyperthyroidism (n = 48); and the hypothyroidism group: those who were diagnosed and treated for hypothyroidism (n = 61). In hyperthyroidism, 21 patients (43.8%) were treated by oral propylthiouracil, and 11 patients (22.9%) were treated by oral methimazole.

### Clinical information

Demographic data included maternal age, parity, method of conception, and history of maternal disease, including diabetes mellitus (DM), hypertension, and psychiatric disease. In vitro fertilization and embryo transfer and intracytoplasmic sperm injection were defined as assisted reproductive technology (ART). Additionally, the clinical information on the number of babies, gestational age at delivery, birth weight, fetal position at delivery, mode of delivery, Apgar scores at both 1 and 5 min, and pregnancy complications including preterm labor, preeclampsia, LFD, PROM, placenta abruption, placenta previa, and preterm birth was collected as maternal and neonatal outcomes.

Preeclampsia was defined as persistent hypertension (systolic blood pressure ≥140 mmHg and/or diastolic blood pressure ≥90 mmHg)) and proteinuria (>300 mg/24 h) after 20 weeks of gestation. LFD was defined as birth weight below the 10th percentile, according to a sex-specific Japanese neonatal anthropometric chart ^(12)^. Preterm birth was defined as delivery between 22 and 36 weeks of gestation. Gestational ages were calculated on the basis of the last menstrual period and confirmed by crown-rump length when the fertilization date was unknown.

### Subanalysis

We determined whether hyperthyroidism was an independent risk factor of placental abruption. The crude ORs and 95% CIs of placental abruption were calculated for each of the following factors: maternal age, parity, hyperthyroidism, preeclampsia, preterm labor, placenta previa, and LFD. These are known to be associated with placental abruption ^(13), (14), (15), (16), (17), (18), (19), (20), (21)^. Maternal age was analyzed as a continuous variable. The aORs and 95% CIs were calculated using multiple logistic regression analyses, which were performed by adjusting the following variables (*p* < 0.05): hyperthyroidism, preeclampsia, and preterm labor.

The hyperthyroidism group was further divided into two subgroups (Figure 1); the placental-related disease group: those with preeclampsia or placental abruption and the nonplacental-related disease group: those not complicated with preeclampsia or placental abruption. To use available data as many as possible, we also included twins with hyperthyroidism in the placental-related disease group (n = 2) and the nonplacental-related disease group (n = 1) from the total study population (n = 3824). In the hyperthyroidism group, all patients with preeclampsia developed after 34 weeks of gestation, late-onset preeclampsia. To assess the effect of thyroid function, including stimulating hormone (TSH), free T_3_ (FT_3_; triiodothyronine), and free T_4_ (FT_4_; thyroxine), and thyroid-stimulating hormone receptor autoantibody (TRAb) positivity on placentation, these were compared in that two subgroups. However, the hyperthyroidism group partially lacked these data because they were treated at other institutes during pregnancy. In the hyperthyroidism group, 34 patients (70.8%) had data of TSH and FT_4_ at the early gestation, from conception to 15 weeks of gestation, because it is thought to be the period of placental development.

### Statistical analysis

All statistical analyses were performed using the SAS (version 9.4, SAS Institute Inc., Cary, North Carolina, USA) software program and JMP pro 15 (JMP, Tokyo, Japan). Continuous variables are presented as median (range), and *p* values were calculated using the Mann-Whitney *U* test and the Kruskal-Wallis test, in comparison with two and three groups, respectively. Categorical variables are presented as numbers (percentage), and *p* values were calculated by Fisher’s exact test. A *p*-value of <0.05 was considered to be significant.

## Results

Table 1 shows the demographic data of the control (n = 3531), hyperthyroidism (n = 48), and hypothyroidism (n = 61) groups. Regarding conception, a significant difference has been detected among the three groups (*p* = 0.026), and the percentage of ART in the hypothyroidism group (19.7%) was higher than those of the control (8.9%, *p* = 0.010). Nevertheless, there were no significant differences in other baseline characteristics like maternal age, nulliparity, and maternal chronic diseases among the control, hyperthyroidism, and hypothyroidism groups.

**Table 1.** Baseline Characteristics and Pregnancy Outcomes of Pregnancies.

	Control (n = 3531)	Hyperthyroidism (n = 48)	Hypothyroidism (n = 61)	*p* Value
**Baseline characteristics**				
Maternal age, years	33 (15-52)	33 (25-42)	33 (22-43)	0.102
<20 years	33 (0.9)	0 (0.0)	0 (0.0)	
20-35 years	2241 (63.5)	29 (60.4)	37 (60.7)	0.907
≥35 years	1257 (35.6)	19 (39.6)	24 (39.3)	
Nulliparity	1959 (55.5)	31 (64.5)	38 (62.3)	0.262
ART	314 (8.9)	4 (8.3)	12 (19.7)	0.026*
Hypertension	28 (0.8)	0 (0.0)	0 (0.0)	1.00
DM	79 (2.2)	1 (2.1)	1 (3.3)	0.661
Psychiatric disease	190 (5.4)	1 (2.1)	4 (6.6)	0.617
**Pregnancy outcomes**				
Gestatinal weeks at birth	38.7 (22.4-42.6)	39.4 (33.1-41.3)	39.3 (32.6-42.0)	0.015*
Birth Weight (g)	2906 (270-4648)	2950 (1610-3812)	3014 (1226-3714)	0.259
Malposition at delivery	270 (7.7)	1 (2.1)	4 (6.6)	0.421
Cesarean section	1461 (41.4)	17 (35.4)	25 (41.0)	0.737
Emergent cesarean section	590 (16.7)	10 (20.9)	13 (21.3)	0.453
Vacuum or Foreceps	367 (7.6)	7 (14.6)	5 (8.2)	0.178
Apgar Score ≤6 at 1 min	368 (10.4)	2 (4.2)	1 (1.6)	0.020*
Apgar Score ≤6 at 5 min	196 (5.6)	0 (0.0)	0 (0.0)	0.031^*^
Preterm labor	376 (10.7)	5 (10.4)	6 (9.8)	1.000
Preeclampsia	156 (4.4)	5 (10.4)	3 (4.9)	0.124
LFD infants^‡^	340 (9.7)	6 (12.5)	5 (8.3)	0.708
PROM	148 (4.2)	1 (2.1)	3 (4.9)	0.796
Placenta previa	149 (4.2)	1 (2.1)	0 (0.0)	0.264
Placenta abruption	16 (0.5)	2 (4.2)	0 (0.0)	0.032*
Preterm birth	507 (14.4)	5 (10.4)	3 (4.9)	0.072

Data are presented as number (percentage) and median (range), and *p* values were calculated by Fisher’s exact and Kruskal-Wallis test, respectively. ^‡^Nine (Control) cases and one (Hypothyroidism) case were missing data of LFD as over 42 weeks of gestation at delivery.*Statistically significant. ART, assisted reproductive technology; DM, diabetes mellitus; PROM, premature rupture of membranes; LFD, light for dates.

Pregnancy outcomes were compared among the control, hyperthyroidism, and hypothyroidism groups. No significant differences were observed in the birth weight, fetal malposition at delivery, cesarean section, and vacuum and forceps. However, the incidence of placental abruption was significantly different in the three groups (*p* = 0.032), and that in the hyperthyroidism group (4.2%) was higher than in the control group (0.5%, *p* = 0.024). Although the incidence of preeclampsia was not significantly different among the three groups (*p* = 0.124), the incidence of hyperthyroidism showed a trend to increase when compared with that in the control group (10.4% vs. 4.4%, *p* = 0.062). The numbers of cases with Apgar scores of ≤6 at 1 and 5 min were significantly different among the control, hyperthyroidism, and hypothyroidism groups (*p* = 0.020 and *p* = 0.031, respectively). The percentage of cases with Apgar scores of ≤6 at 1 min in the control group (10.4%) were higher than the hypothyroidism groups (1.6%, *p* = 0.018). Gestational weeks at birth were earlier in the control group when compared with hyperthyroidism and hypothyroidism groups (*p* = 0.015). The incidence of preterm birth in the control (14.4%) was also higher than that in the hypothyroidism (4.9%) groups (*p* = 0.040), but no significant difference was detected among the three groups (*p* = 0.072). The differences in the other incidences, such as preterm labor, LFD infants, PROM, and placenta previa among the control, hyperthyroidism, and hypothyroidism groups, were insignificant.

Other variables were also examined to determine whether hyperthyroidism was an independent risk factor of placental abruption (Table 2). Factors found to increase placental abruption included hyperthyroidism (crude odds ratio, OR = 9.71, 95% confidential interval, CI: 2.17-43.48), preeclampsia (crude OR = 4.30, 95% CI: 1.23-15.00), and preterm labor (crude OR = 3.26, 95% CI: 1.15-9.20). After multivariate analysis, hyperthyroidism (adjusted OR, aOR = 8.21, 95% CI: 1.76-38.34), preeclampsia (aOR = 4.10, 95% CI: 1.13-14.76), and preterm labor (aOR = 3.38, 95% CI: 1.19-9.64) were found to be independently associated with an increased risk of placental abruption.

**Table 2.** Univariable and Multivariable Logistic Regression Analysis of Factors Potentially Associated with Placental Abruption.

	Univariable analysis			Multivariable analysis		
	Crude OR	95%CI	*p* value	aOR	95%CI	*p* value
Maternal age (years)	0.957	0.869-1.049	0.357
Nulliparity	0.793	0.314-2.00	0.625
Hyperthyroidism	9.71	2.17-43.48	<0.01*	8.21	1.76-38.34	<0.01*
Preeclampsia	4.30	1.23-15.00	0.022*	4.10	1.13-14.76	0.031*
Preterm labor	3.26	1.15-9.20	0.025*	3.38	1.19-9.64	0.023*
Placenta previa	1.37	0.18-10.37	0.760
LFD infants	2.68	0.88-8.21	0.083

Maternal age is analyzed as a continuous variable: Its odds ratio is the odds ratio for each additional year of age. OR, odds ratio; CI, confidence interval; aOR, adjusted OR; LFD, light for dates. * Statistically significant.

**Figure 2.**
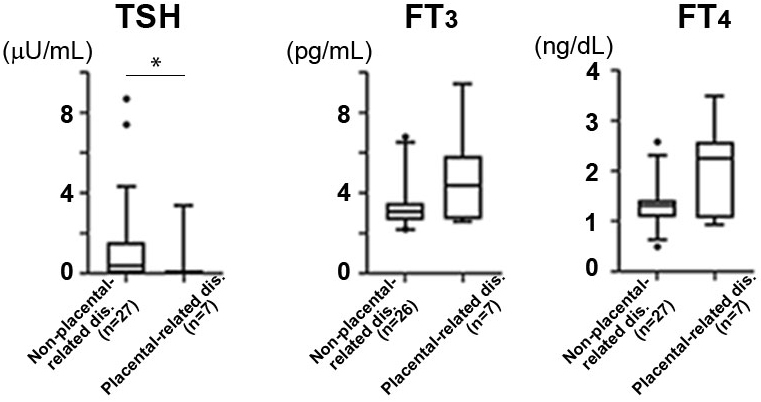
The thyroid function at early gestation was compared between the nonplacental-related diseases and placental-related disease groups. Nonplacental-related dis.: successful placentation group, those who had no adverse pregnancy outcomes. Placental-related dis.: those who had preeclampsia or placental abruption. Median data [5%-95%] is shown. *p < 0.05.

We examined thyroid function at the placentation period, early gestation, to detect specific features in hyperthyroidism that developed placental abruption and preeclampsia. Placental abruption is known to have common pathology as placental-related diseases with preeclampsia ^(22)^. In the hyperthyroidism subgroup, serum TSH levels in the placental-related disease group were significantly lower than those in the nonplacental-related disease group (*p* = 0.043, Figure 2). Although FT_3_ and FT_4_ were higher in the placental-related disease group, the difference was insignificant (Figure 2). When comparing TRAb positivity values, no significant difference was detected between the two groups (placental-related diseases―6/8; 75.0%, nonplacental-related diseases―16/28; 57.1%, *p* = 0.441).

## Discussion

In the present study, pregnancy outcomes in women with hyperthyroidism and hypothyroidism under treatment were comparable with those without thyroid disease. However, the present study revealed that hyperthyroidism had a significantly higher incidence of placental abruption and a trend for increased preeclampsia when compared with the control. Moreover, multivariate analyses showed hyperthyroidism, preeclampsia, and preterm labor as possible risk factors for placental abruption. Additionally, in the hyperthyroidism group, serum TSH levels at the placentation period were lower than in the placental-related disease group, including preeclampsia and placental abruption, and nonplacental-related disease group.

Similar to the present data, hyperthyroidism is associated with a high risk of preeclampsia^(5), (6), (7), (8)^ and placental abruption ^(9)^. Additionally, a previous study reported a higher occurrence of hypertensive disorders of pregnancy in the treated group than in control ^(11)^, which was consistent with the result in this study because preeclampsia is included in hypertensive disorders of pregnancy.

Additionally, the present study revealed that the maternal and neonatal outcomes in women with treated hypothyroidism were similar to those without thyroid disease. Both overt hyperthyroidism and hypothyroidism are well known for adverse effects on the mother and her child ^(23)^. Additionally, ART is known to be associated with increased adverse pregnancy outcomes ^(24)^. However, pregnancy outcomes were similar in this study between hypothyroidism and control groups despite a higher prevalence of ART in hypothyroidism. This result is consistent with a previous report ^(3)^ and suggests that treatment of hypothyroidism could decrease the risk of adverse pregnancy outcomes seen in overt hypothyroidism.

The recent large cohort reported that the prevalence of placental abruption is as low as 0.5% in Japan ^(21)^, which is consistent with the present study. Maternal age, parity, ART, hypertension, DM, psychiatric disease, preeclampsia, preterm labor, LFD, PROM, and placenta previa are well known to be risk factors for placental abruption ^(13), (14), (15), (16), (17), (18), (19), (20), (21)^. The present study also showed that preeclampsia and preterm labor were significantly increased in the placental abruption group. However, no significant differences were shown in the other risk factors in the present study. The inconsistency would be related to the small number of the placental abruption group. Multivariate analysis demonstrated that hyperthyroidism, preeclampsia, and preterm labor were independent risk factors for placental abruption in the present study. Several retrospective cohort studies have also reported the association between placental abruption and hyperthyroidism ^(9), (25)^. However, whether treatment for hyperthyroidism could influence the risk of placental abruption remains unknown because the present study could not include a population of untreated hyperthyroidism. Furthermore, as randomized controlled trials lacked, the impact of antithyroid interventions for hyperthyroidism prepregnancy or during pregnancy on important pregnancy outcomes remains unknown ^(9)^.

Preeclampsia and placental abruption are recently thought to be associated with common pathology as placental-related diseases due to inefficient invasion of trophoblasts after implantation ^(22)^. The patients with preeclampsia in the subanalysis were late-onset preeclampsia, and shallow placentation is also detected in late-onset preeclampsia ^(26)^. Human chorionic gonadotropin from the trophoblast stimulates thyroid hormones at early gestation. Thyroid hormones play a role in placentation, which is completed at almost 20 weeks of gestation ^(27)^, and T_3_ is known to play a critical role in placentation by increasing trophoblasts invasion ^(28)^. These findings led us to hypothesize that thyroid subdysfunction at the placentation period might cause placental-related diseases in hyperthyroidism. To confirm it, we examined TSH levels during the placentation period. They were significantly lower in the placental-related disease group than the nonplacental-related disease group among patients with hyperthyroidism. Serum FT_3_ and FT_4_ levels in the placental-related disease group were higher than those in the nonplacental-related disease group, but the difference was insignificant. This thyroid status at the placentation period in the placental-related disease group might be similar to subclinical hyperthyroidism. The previous studies reported the association of placental abruption with subclinical hyperthyroidism at early gestation ^(25), (29)^, consistent with the present study. Therefore, these results suggested that treated hyperthyroidism who showed subclinical hyperthyroidism at the placentation period might cause placental-related diseases, including preeclampsia and placental abruption, although further investigation is required. Thus, close check-ups for thyroid function might be concerned at this period in hyperthyroidism. The occurrence of placenta abruption is approximately 0.4% in Japan ^(30)^. Therefore, larger cohort is needed to prove it.

### Limitations

The main limitations of this study consisted of the study design being a cohort study and the inclusion of a single tertiary care institution. Prevalence of low Apgar scores and higher preterm births, as well as significantly earlier gestational weeks at birth in the control group when compared with the thyroid disease groups, suggests that the study population belonged to a high-risk category for adverse pregnancy outcomes. Additionally, the general occurrence rate of placental abruption was also low, that is, 0.5%-1.0%. However, the multivariate analysis of the risk of placental abruption might eliminate bias by accounting for confounding factors. Second, all women in the control group were not screened for thyroid dysfunction. Hence, a small but significant proportion of these women could have undiagnosed subclinical thyroid dysfunction, influencing the study results. Subclinical hypothyroidism was associated with preterm birth ^(7)^, and these patients may have been included in the control group. Additionally, the information on the time to start treatment, the duration of treatment, and treatment adherence was also lacking, because some of the patients were treated at other hospitals before pregnancy and clinical information before pregnancy was limited. Data on thyroid function were also unavailable for the patients, followed up by other clinics. Most patients treated with methimazole before conception were transitioned into propylthiouracil (n = 7) or reduced (n = 5) during early pregnancy (data not shown). This treatment alternation might cause transient instability of thyroid function but could not be analyzed because of missing data. Further study is needed on the risk of alternation of medication during pregnancy in hyperthyroid patients. Moreover, the sample size for the comparison between the placental-related diseases and nonplacental-related disease groups in hyperthyroidism was small. Further prospective research with a large study population should be conducted on the effect of the intervention before pregnancy on pregnancy outcomes to determine the adequate management of women with hyperthyroidism who wish to be pregnant.

### Conclusion

The risks of pregnancy outcomes among women with treated thyroid diseases are comparable with those without thyroid diseases. Although further research is required in a large study population to validate, hyperthyroidism might be an independent risk of placental abruption.

## Article Information

### Conflicts of Interest

None

### Sources of Funding

This work was supported by JSPS KAKENHI (Grant Number: 15H02660).

### Acknowledgement

We would like to thank Editage (www.editage.jp) for the English language editing.

### Author Contributions

TK contributed to the conception and design of the study. TK, KI, TU, YM, TNK, HT, and SS performed the acquisition and interpretation of data and revised it critically for important intellectual content. AH and F Kinoshita, as biostatisticians, contributed to the analysis of the data. TK drafted the first version of the manuscript. SO, EY, AI, F Kikkawa and HK contributed to interpreting the data and revising it critically for important intellectual content. All authors gave their approval for the final version of the manuscript.

### Approval by Institutional Review Board (IRB)

The study was approved by the ethics committee of Nagoya University Graduate School of Medicine (2015-0415, 2017-0414).
